# Rapid ILs-polishing Processes Toward Flexible Nanostructured Paper with Dually High Transparency and Haze

**DOI:** 10.1038/s41598-017-07008-y

**Published:** 2017-07-31

**Authors:** Yanghao Ou, Jinbo Chen, Pengbo Lu, Fan Cheng, Meiyan Lin, Lingfeng Su, Jun Li, Detao Liu

**Affiliations:** State Key Laboratory of Pulp and Paper Engineering, South China University of Technology, Guangzhou, 510640 China

## Abstract

Biodegradable highly nanostructured paper has received great interest in past years due to its excellent optical properties which facilitate its wide applications in green flexible electronics and devices. However, energy and/or time-consuming procedure during the process of fabricating most nanostructured transparent paper are presently the main obstacle to their scalable production. In this work, we demonstrated a novel nanostructured paper with dually high transparency (∼91%) and high haze (∼89%) that was directly fabricated from original paper with rapid ILs-polishing processes. The whole fabricating time only requires 10 min. Compared to the previously reported nanopaper made of the isolated cellulose nanofibers by pure mechanical and/or chemical approaches, this work presented herein is devoted to use green ILs to polish directly the micrometer-sized fibrous paper into the nanostructured paper. This new method brings a rapid fabrication of transparent nanostructured paper while also retaining dual intriguing properties both in optical transmittance and haze. This work is capable of fabricating next-generation flexible and highly transparent and haze paper by a high-speed roll-to-roll manufacturing process with a much lower cost.

## Introduction

Nanostructured transparent paper, as an alternative to glass, plastic, and silicon wafer, is a hotspot among scientific communities in worldwide due to its unique optical and mechanical properties. In past years, many publications were reported to enlarge its applications in flexible electronic devices such as solar cells^1, 2^, transistors^3, 4^, organic light-emitting diodes^5^, touch screens^6^, and antennas^7^. These cases state its superiorities compared to the commercial plastics-, glass- or metal-based substrates. Currently, much as the nanostructured transparent paper mostly attained by assembling the separating cellulose nanofibers resulted from the appropriate pure mechanical^8^, enzyme^9^, acid^10^ and/or TEMPO-oxidized pretreatments^11–13^. Yet, these methods were mostly found to be high energy and/or time-consumed, which hinders their further large scale for industrialization. Other biodegradable transparent papers or cellulose films were also reported to be fabricated by casting or extruding of cellulose by complete dissolution in solvents of such DMAC/LiCl^14, 15^ and ILs^16, 17^, however, incorporating massive cellulose solvents into cell wall of wood fibers by completely dissolution of cellulose brings great waste of solvents, time-consumed, and also adds cost. Surface selective dissolving approach^18^ was recently reported to enhance mechanical and optical properties of the all-cellulose composites, but did not required the complete dissolution of cellulose. For example, the original papers fabricated from bacterial cellulose fibers^19^, lyocell fibers^20^, wood fibers^16^ and ramie fibers^18^ were firstly immersed in 8% DMAc/LiCl for 2~12 h at room temperature for the surface partial dissolution of cellulose. This part dissolution of cellulose fibers was demonstrated to greatly improve the elastic modulus and tensile strength of the composites; however, the optical transparency is still low and maintains a translucent level^16^. Compared to the reported DMAc/LiCl solvent, ILs was demonstrated to have a great superiority in dissolving ability of cellulose^21^. The previous works were reported to immerse the commercial filter paper, and also those based on nanofibrillated cellulose (NFC) in BminCl ILs at 80~85 °C^22^. Although the transparent paper has highest transparency of ~76% (800 nm)^22^, but still remains a low level^18^ which does not meet the optical requirements for the substrates on optoelectronic devices.

In this work, we demonstrated a novel ILs-polishing method to rapidly fabricate a highly nanostructured transparent paper directly from an original paper, which has the dually high transparency (~91%) and high haze (~89%) at 550 nm. ILs is rapidly brushed onto the double-side of original paper and infiltrates the cell wall of cellulose fibers, which provides the chance for facilitating the part surface dissolution of cellulose matrix. The ILs-polishing process is completed after firstly regenerating and subsequently drying for the resulting cellulose matrix, and further repeated by three times to obtain the satisfied optical properties. Nanostructured transparent paper in our work has the following superiorities over the previously reported transparent papers: (1) Rapid ILs-polishing process can be directly used to fabricate nanostructured paper dually with high transparency and high haze; (2) total time of ~90 s to ILs-polish the original paper shows a magnitude decrease compared to those reported transparent paper. It’s highly nanostructure and imporous is responsible for its outstanding optical and mechanical properties, and the rapid ILs-polishing process makes possibly viable route towards the fast production of nanostructured transparent paper by the roll-to-roll manufacturing process^23^.

## Results and Discussion

**Figure 1:**
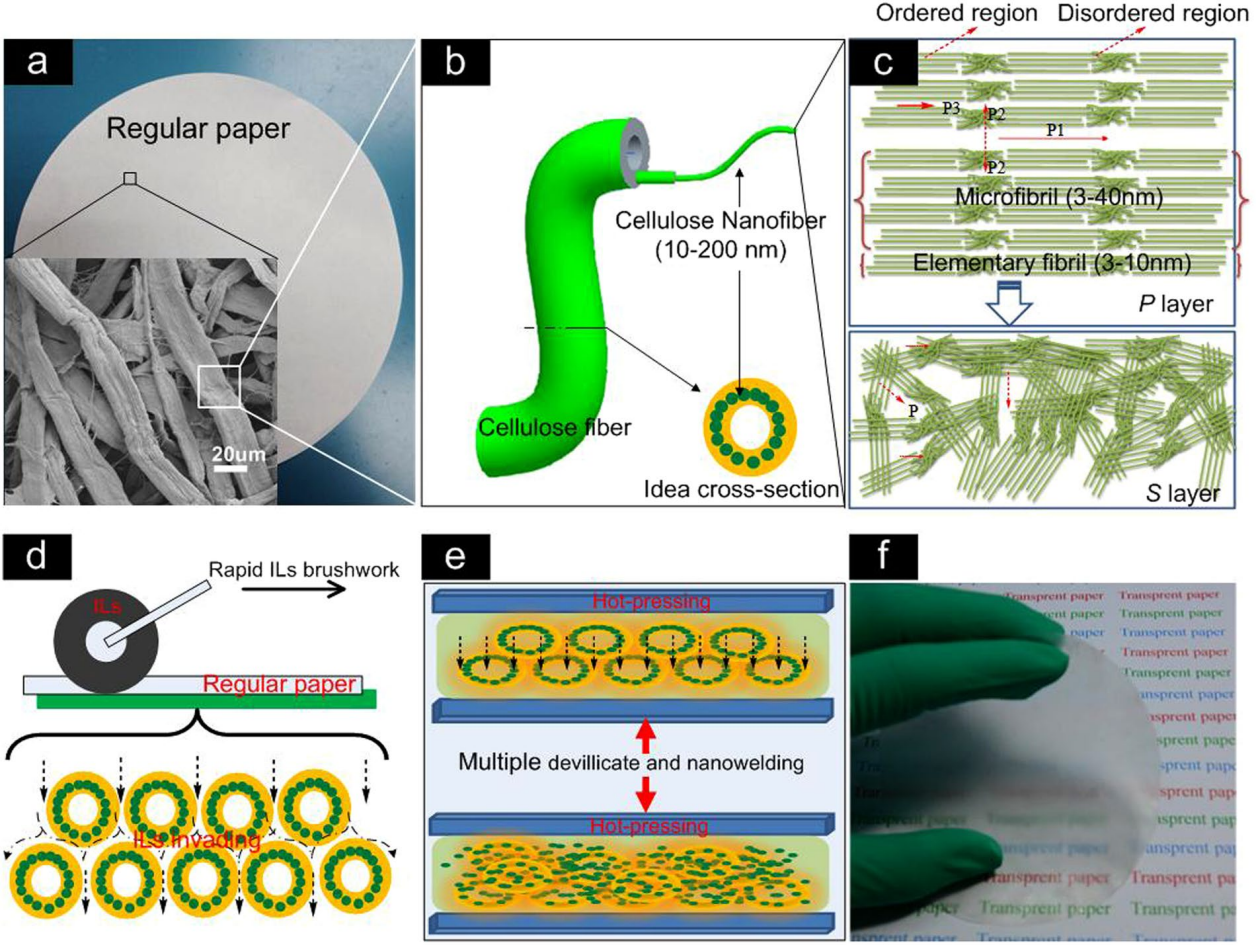
Schematic of the rapid ILs-polishing process for directly fabricating the nanostructured transparent paper from a regular paper. (**a**) Regular paper. The insert in the bottom left corner is a SEM image of the regular paper. (**b**) The ideal structure of single cellulose fiber. The insert in the bottom right corner is a magnified idealdiagram of cellulose nanofibers distributing along the cross section of a single cellulose fiber. (**c**) The various invading pathways of ILs in the P, L layers on the cell wall. The *S Layer* indicates the invading channel for ILs in disordered *S* region, while the *P1*, *P2*, *P3* indicates respectively the invading channel for ILs exiting in the ordered region of gaps microfibrils, amorphous and elementary fibrils. (**d**) The invading models of ILs in fibrous cellulose matrix under a hot pressing process. (**e**) Formation of cellulose nanofibers and its nanowelding process. (**f**) Digital picture of the nanostructured paper.

A schematic diagram showed the evolution in the hierarchical structure of the original paper to the nanostructured transparent paper by ILs-polishing method (Fig. 1). The original paper based on micrometer-sized cellulose fibers and voids shows opacity owing to the difference in refractive index between cellulose and air in voids. Void between or/and inner cellulose fibers ranges from nanoscale size to microscale size (Fig. 1b,c). When the air in voids is completely removed or replaced by cellulose, the resulting paper becomes transparent due to the uniformity of the refractive index along the whole paper. EmimMeOPO_2_H ILs synthesized in this work is composed of an organic cation with an inorganic counter ion, and has the strong dissolving ability for cellulose. When rapidly brushing ILs onto each side of the original paper, the ILs preferentially fills in the voids between cellulose fibers and subsequently permeates into the cell wall of cellulose (Fig. 1d). Total four invading pathways of ILs into the cell wall of cellulose include respectively the gaps in the secondary wall (*S* layer, *P* gap), and gaps neighboring the microfibrils (*P*_*1*_ gap) and even nanofibrils (*P*_*3*_ gap) as well as the amorphous region (*P*_*2*_ gap) (Fig. 1c). After completing the invasion process, the resulting paper was performed with a hot-pressing process which facilitates the physical interaction between ILs and cellulose. A large amount of hydroxyl groups in cellulose molecule enables facile formation of hydrogen bonds and highly ordered structure which is responsible for its desirable mechanical properties. The oxygen and hydrogen atoms of the cellulose form electron donor-electron acceptor complexes with the charged species of the ionic liquids. For example, cations from EmimMeOPO_2_H ILs easily connect to oxygen in the inter- and intramolecular H-bonds between cellulose chains, whereas the anions connect to the hydrogen atom. This process breaks the inter- and intramolecular hydrogen bonds of cellulose chains, leading to the dissolution of cellulose fiber^23^. By controlling the ILs addition as well as the ILs-polishing process, the surface of cellulose fibers is partly dissolved without completed dissolution (Fig. 1d). The compulsive pressure resulted from the hot-pressing process causes the collapse of the cell wall of cellulose, and facilitates the part surface dissolution of cellulose fibers (Fig. 1e). In this hot-pressing process, the dissolved cellulose solution as well as the hot EmimMeOPO_2_H ILs further rapidly infiltrates into voids between/inner the cell wall of cellulose, enabling formation of the nanostructured cellulose nanofibers and its reconstruction of cellulose matrix. By regenerating in ethanol, the EmimMeOPO_2_H ILs was replaced by ethanol in which the partly dissolved cellulose in the voids or/and on the surface of cellulose fibers was instantaneously gelled to bond the neighboring cellulose nanofibrils together. As a result, most of the air in voids was replaced by cellulose which results in a compact density and the uniform refractive index along the whole thickness of the nanostructured paper. ILs-polishing by hot-pressing the resulting paper yielded them with perfectly optical and mechanical performances (Paper-1). Once the first ILs-polishing process had elapsed, the resulting paper was further treated by the same ILs-polishingprocess with the second time (Paper-2) and the third time (Paper-3), rendering the optical properties of the nanostructured paper with dually high transparency and haze (Fig. 1f). An important result in this regard was that ILs-polishing appears to be an ideal pathway for the part surface dissolution of cellulose, being both rapid and controllable.

**Figure 2:**
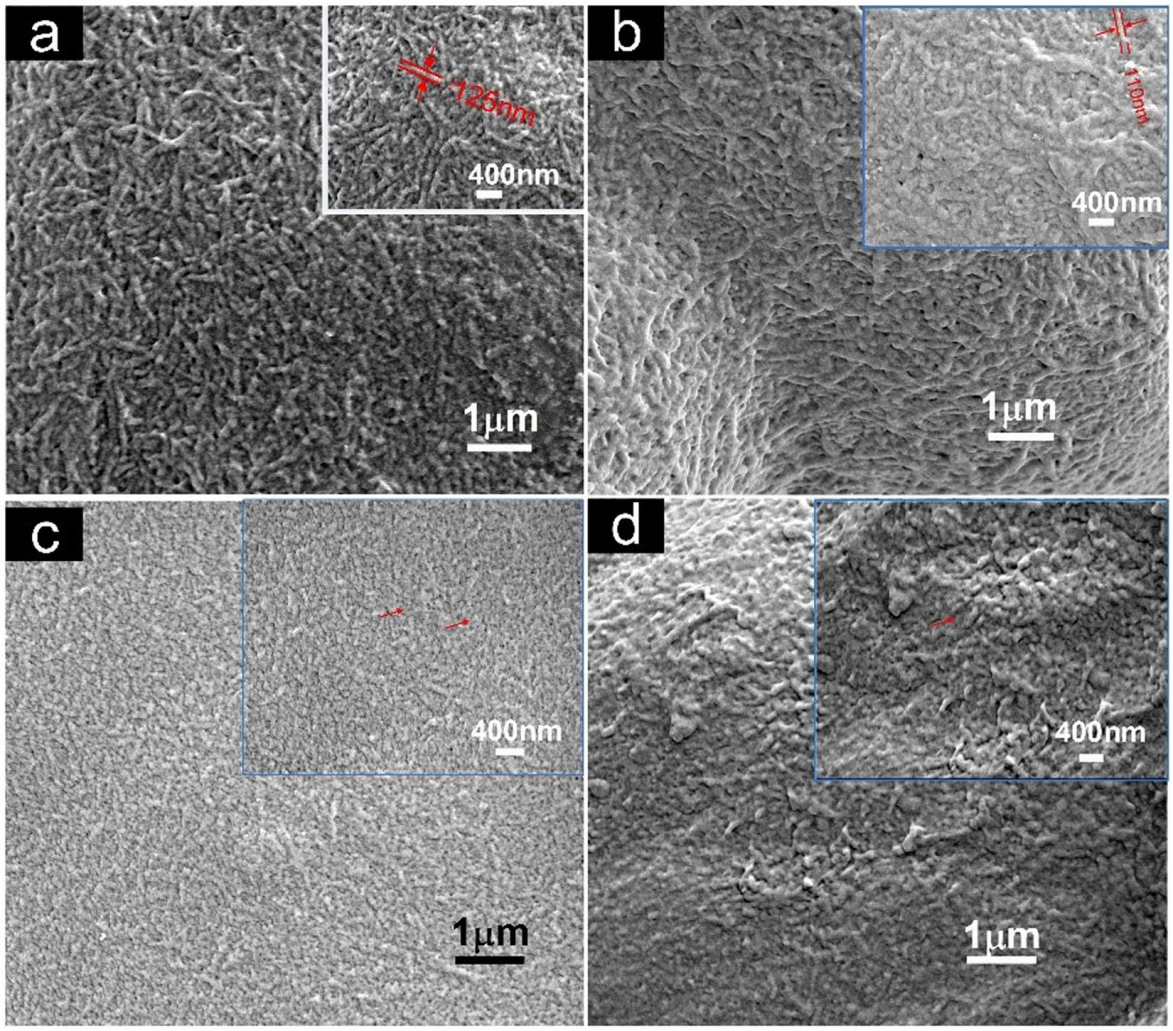
SEM images of evolution in the morphological structure of cellulose nanofibers after hot-pressing by 5 min (**a**), 10 min (**b**), 15 min (**c**) and 60 min (**d**).

Figure 2 illustrates the evolution in formation and morphology of cellulose nanofibers as a result of time in the first ILs-polishing process. It was found that the uniformly distributed cellulose nanofibers with 125 nm diameter were clearly observed by ILs-polishing for 5 min. Increasing time to 10 min decreases the diameter of cellulose nanofibers to about 110 nm. Studies utilizing both ILs and polishing process that replace the typically chemical or mechanical separation of cellulose nanofibers as well as the total dissolution of cellulose have suggested that the ILs-polishing at mild conditions could be effective. After further ILs-polishing them to 15 min, the dramatic dissolution of cellulose fibers resulted in near disappearance of the basic profile of cellulose nanofibers, indicating the excessive dissolution of cellulose. However, cellulose nanofibers nearly disappear after a 60 min hot-pressing, and the cellulose nanofibers were completely dissolved without any fibrous profiles.

**Figure 3:**
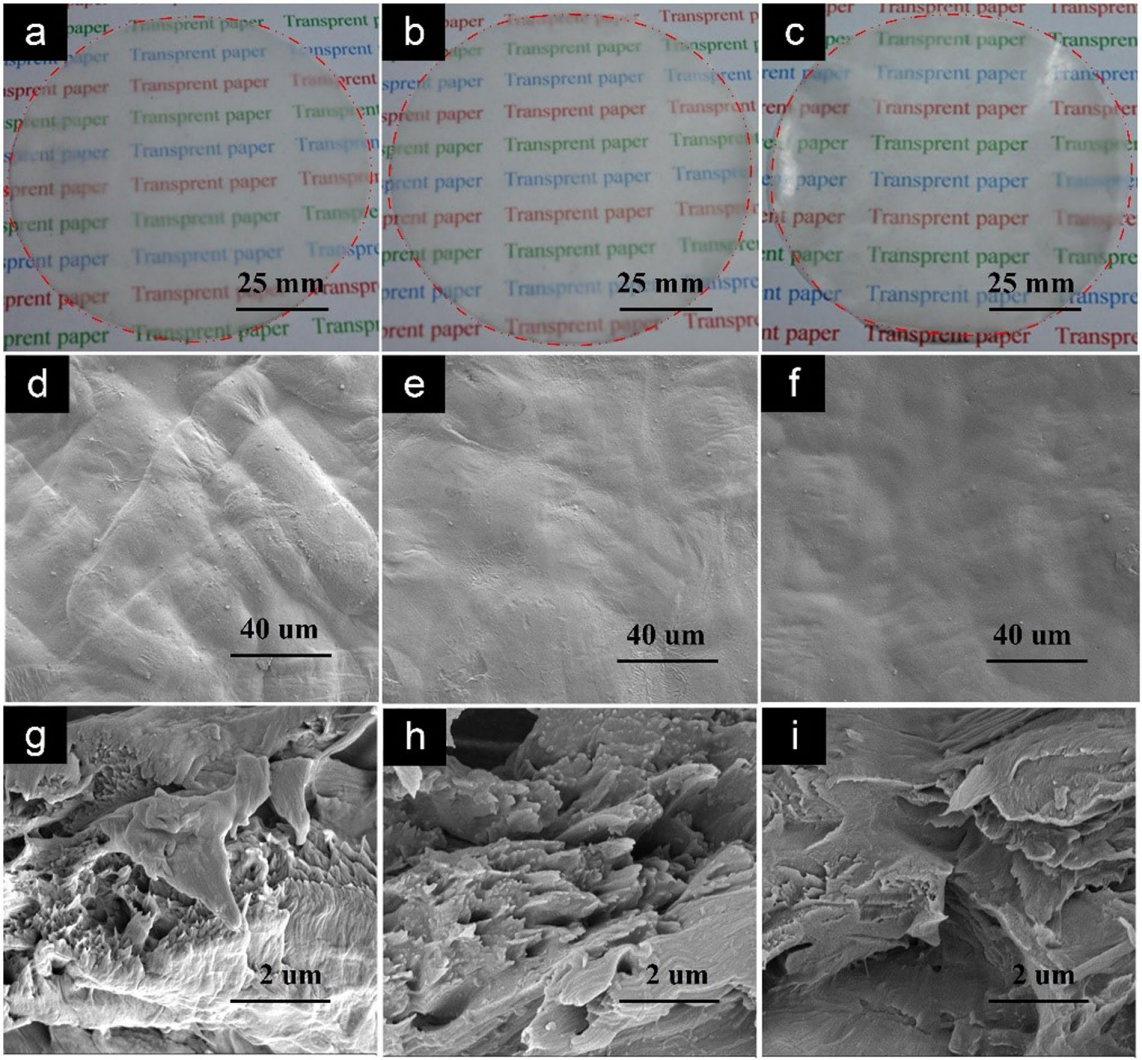
Digital images of nanostructured transparent paper prepared with the repetitious ILs-polishing process by the first (**a**), the second (**b**) and the third time (**c**) respectively. SEM images of the surface (**d**–**f**) and the cross-section (**g**–**i**) of resulting paper responding to the ILs-polishing processes.

In the reported partly dissolving approaches of cellulose^19–22^, much longer time is used to immerse the all-cellulose matrix in ILs or DMAc/LiCl by one time, however, the optical transparency of the resulting all-cellulose samples is much lower due to the retained larger cellulose fibers along the whole thickness. As the demand for highly transparent paper continues to grow, the repetitious ILs-polishing process was firstly reported in our work to fabricate highly transparent paper. However, the profile of larger cellulose fibers but not the voids between the neighbouring cellulose fibers was still clearly observed when only polishing ILs onto the surface of the regular paper by the first time (Fig. 3a,d,g). When the above ILs-polishing process was repeated for the second time, the surface of cellulose fibers smoothed without obvious fibrous profile (Fig. 3b,e,h). This can be speculated to result from that the ILs-polishing enables the more surface dissolution of cellulose fibers which gradually peels the excrescent cellulose from the concavo-convex fibrous matrix. After polishing the surface of the regular paper with ILs by the third time, the fibrous profile of cellulose fibers entirely disappeared without any porosity which provides a smooth surface for the resulting nanostructured paper (Fig. 3c,f,i).

**Figure 4:**
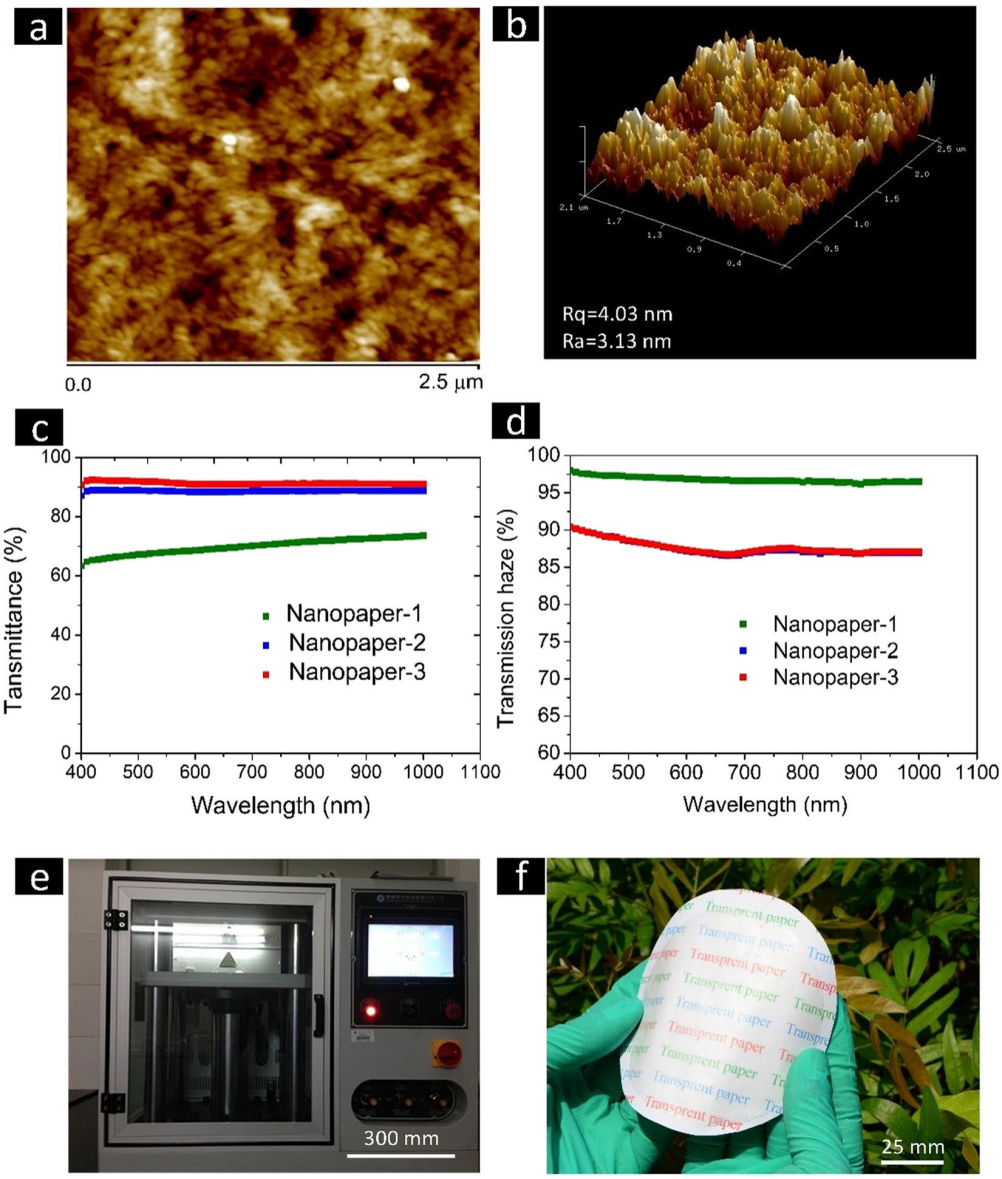
Surface morphology of the nanostructured paper is studied by AFM for 2-D height image (**a**) and 3-D height image (**b**) at the scan size of 2.5 μm × 2.5 μm. Optical transmittance curve (**c**) and optical haze (**d**, 550 nm) of the different nanostructured paper samples ranging from 400 nm to 1100 nm were compared. Digital images of the experimental equipment (**e**) and the fabricated nanostructured paper with dually high tranparency and haze (**f**).

A primary and underlying question is whether surface roughness of flexible transparent substrates from various sources meets the requirements in fabricating flexible devices when printing nanometer-sized electronic components onto the flexible substrates. For this reason, the work presented herein is devoted to overcoming the potential challenges of higher surface roughness presents in fabricating flexible fibrous substrates. As can be seen in the Fig. 4a and b, the surface of the nanostructured paper was scanned by AFM technology, investigating the 3D profile and also surface roughness. When the AFM scanning range is 2.5 μm, the surface roughness of the nanostructured paper is 4.03 nm (Rq) and 3.13 nm (Rq), which has a comparatively low surface roughness to the reported typical nanopaper (Fig. 4b). This much low roughness provides the basis for directly printing active nanometer-sized electronic components on the nanostructured paper in future manufacturing.

The optical properties of the nanostructured paper were performed with a UV spectrophotometer. The optical transmittance of the nanostructured paper for the first ILs-polishing process was about 70% at 550 nm, which agrees well with the previous report^22^. When repeating the ILs-polishing process by the second and third time, the optical transmittance of the nanostructured paper increases to 90%, 91% (at 550 nm), respectively (Fig. 4c). The optical transparency of the nanostructured nanopaper is much higher than those surface partly dissolved paper with the highest transparency of ~76% (800 nm), and maintains the equivalent level compared to the nanopaper^5, 24, 25^ made of the chemically separated cellulose nanofibers. More importantly, the nanostructured paper also has the outstanding optical property of high haze because the partly dissolved cellulose binds individual sheets together while maintains the larger fibrous profiles. When firstly ILs-polishing the original paper, the haze of the nanostructured paper has the highest haze value of ~94% while the optical transparency maintains a lower value of about 70% (Fig. 4c,d). The haze value of the nanostructured paper decreases to 85% when the optical transparency increases to 90% after the second ILs-polishing process. The optical transparency and haze of the nanostructured paper maintain slight changes when continuing the third ILs-polishing process. The observed trade-off between the optical transparency and haze properties were also demonstrated by Larsson’s work which uses the oxidized cellulose fibers with a simple papermaking procedure^23^. It has comparatively optical properties similar to the previously reported highly transparent and hazy paper^23, 26^, but only requires several times decrease in preparing time. Alternatively, the rapid ILs-polishing process is an efficient process to fabricate the nanostructured paper that displays dually high optical transparency (∼96%) and high haze (∼89%) that will continue to be developed in future a large scale and at low cost combined with roll-to-roll manufacturing processes. The attractively optical properties make the nanostructured paper ideal candidate materials for high-impact applications in numerous fields, such as flexible solar cell devices.

Repeating the ILs-polishing process from one to three time yielded the resulting paper with highly optical transparency and surface smoothness (Fig. 4f).

**Figure 5:**
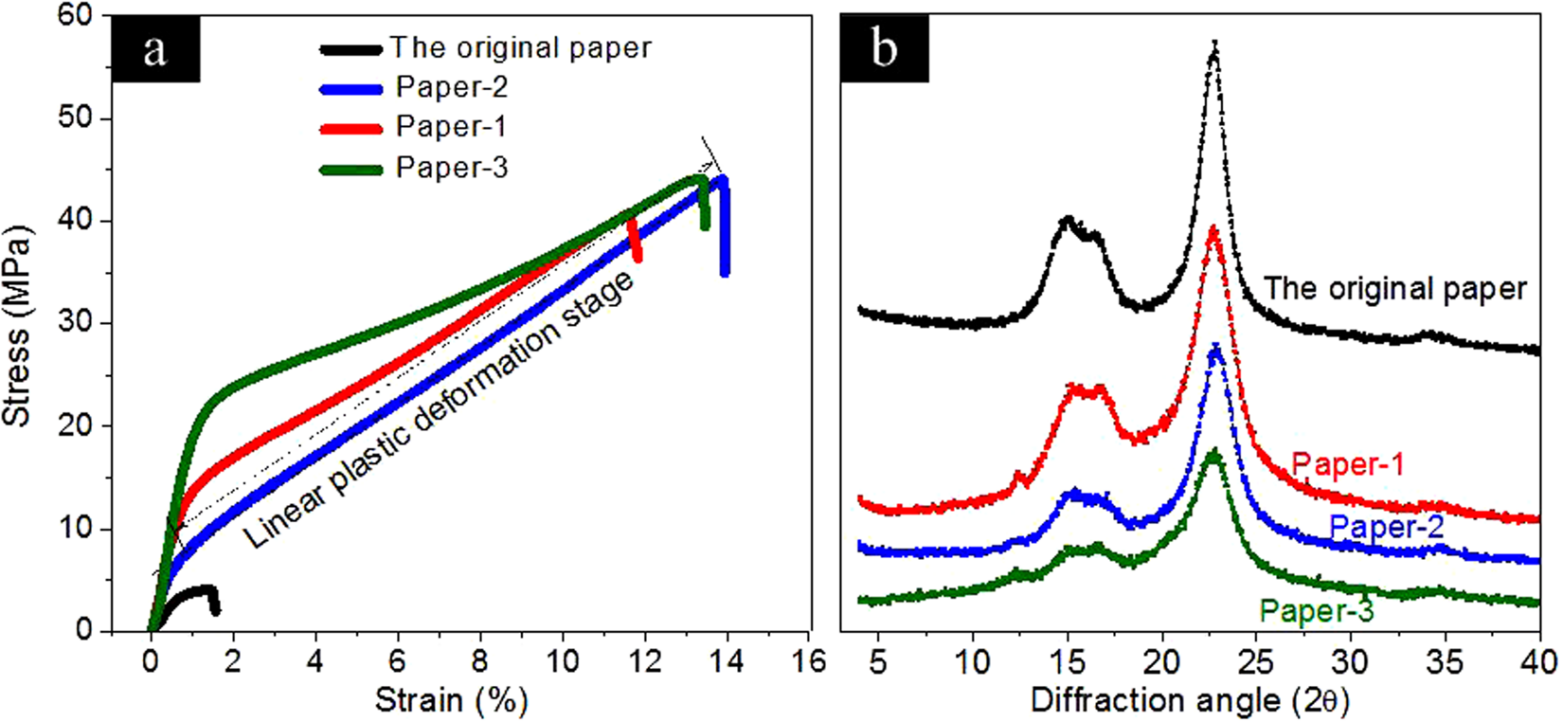
Stress-strain curves (**a**) and XRD curves (**b**) of the Paper-1, Paper-2, Paper-3 and original paper samples.

Figure 5 showed the XRD curves of the original paper, as well as the nanostructured transparent paper resulted from the first (Paper-1), the second (Paper-2) and the third (Paper-3) ILs-polishing process. It was found that the main diffraction signals were 14.86°, 22.75°, which are normally assigned to the diffraction planes 101, 002 respectively. As can be shown in the Fig. 5b, The original paper has proportional XRD pattern with the typical cellulose I^27^. The major diffraction intensities for the nanostructured paper by the ILs-polishing process observed around at 14.86°, 22.75° were less intense. It was also observed that there were no cellulose II diffraction peaks in XRD pattern after the ILs-polishing process with three times. This can be speculated to result from that the basic preserve of the originally fibrous profile in nanostructured transparent paper without excessive dissolution of cellulose, but only by repeatedly ILs-polishing the surface of cellulose fibers. Under this condition, the partly dissolved cellulose peeled from the surface on the cellulose fibers fills in the voids with a small amount of ILs and few time, being much few than those reported by immersing in ILs for longer time with large ILs^5^.

The mechanical strength of the original paper, Paper-1, Paper-2 and Paper-3 samples was performed with a tensile testing machine. As can be seen in Fig. 5a, the strength of the original paper is comparably low than those samples by ILs-polishing, and has a wider range of strain. This can be speculated to result from the partly surface-dissolved cellulose generated from the first ILs polishing procedure is regarded as a promising material that could replace hydrogen bond and bond tightly the resulting cellulose fibers together^28^. Meanwhile, the remaining fibers remained largely unaffected and hence maintain most of their initial structure and strength. The strength of transparent paper samples maintains slight changes, and Paper-3 samples has the best tensile strength after the third ILs-polishing. In addition, larger amounts of fibrous matrix are formed since larger fractions of the fibers are dissolved when the times of ILs-polishing continue to increase. It was also observed that the stress-strain curve shows good linear relationship responding to the Paper-2 samples, which indicated the characteristics of plastic deformation by controlling the ILs-polishing process and its conditions in future experiments.

## Conclusions

In conclusion, this work provides the demonstration that directly rapid ILs-polishing for disintegrating *in situ* the cellulose fiber dimensions from micrometer-sized level into nanometer-sized level enables the flexible paper with dually high transparency and haze. We experimentally investigate the relationship between the ILs-polishing processes and optical properties of the nanostructured paper. It was concluded that the dimension size of cellulose nanofibers derivate from the mesoporous fiber structure was controlled by varying the ILs-polishing conditions. The nanostructured transparent paper has high transmittance value of ~91% and haze value of ~89% at the visible light wavelength of 550 nm respectively. The low surface roughness with 4.03 nm (Rq) of our transparent paper provides good chance for printing nanometer-sized electronic components. More importantly, compared to the optical property of the typical nanopaper, glass and plastics, our nanostructured transparent paper are of great interest in flexible photovoltaic power devices with roll-to-roll manufacturing because the dually high transparency and haze with acceptably short processing period.

## Experimental

### Synthesis of EmimMeOPO_2_H

Dimethyl phosphite and 1-Methylimidazole were used to synthesize EmimMeOPO_2_H ILs in Ar atmosphere, and the synthesis procedure was performed at the previously reported literature^29^.

### Fabrication of the nanostructured paper

The dissolving grades chemical wood pulp contains more than 90% cellulose, which were used as the basic material for fabricating the original paper. The original paper and EmimMeOPO_2_H ILs were dried for at least 24 h in the vacuum drying oven at 80 °C before the experiments. 2 ml of ILs was quickly coated on each side of the original paper by a brush at room temperature and a dried environment. The resulting paper was then hot-pressed on the BL-6170-B mode machine at 100 °C with 1.0 Mpa. After completing the hot-pressing process, the treated paper samples were regenerated in the ethanol to remove the residual ILs, and then placed in the BL-6170-B mode machine again at 100 °C with 1.0 MPa for a rapid drying process. Above mentioned ILs-polishing process is repeated by three times to further polish the original paper and the nanostructured transparent paper was obtained.

### Characterizations

The sheet surface and fracture surface of the nanostructured transparent paper and original paper were observed using a Scanning Electron Microscopy (Zeiss Merlin), with the acceleration voltage of 10–15 kV. All the samples were fixed to a metal-base specimen holder by double-sided conductive tapes and coated with gold.

The crystal structures of the nanostructured transparent paper and original paper were detected using a D8 ADVANCE (Bruker Co.) XRD analyzer, at 40 kV and 30 mA. The scanning rate was 12°/min and step increment was 0.04°. The diffraction angle of 2θ ranging from 4–50° was recorded for wide-angle x-ray intensities.

The degree of crystallinity is calculated by the following equation^30^:

1 $${X}_{c}=({I}_{c}-{I}_{(am)})\times 100/{I}_{c}$$

where *X*_*c*_ indicates the crystallinity of BKSP sample; *I*_*am*_ is the intensity of amorphous regions of 001 (Cellulose I) and 002 (Cellulose II) at diffraction angle of 15.0°and 18.0°, respectively; and *I*_*c*_ is the intensity of crystal planes of 001 (Cellulose I) and 002 (Cellulose II), respectively.

The crystallite size of BKSP sample is calculated by the Scherrer formula^31^:

2 $$D=K\lambda /\beta \,\cos \,\theta $$

where *K* is the Scherrer constant (0.89), *D* is the apparent crystallite size (nm), *λ* is the wavelength of the X-ray (0.154056 nm), *β* is the full width at half maximum (rad), and *θ* is the diffraction angle.

The tensile properties of the nanostructured transparent paper and original paper were carried out with an Instron 5565 mode universal tensile tester. The dimension of all testing samples is 15 mm-width and 40 mm-length. And a cross-head speed of 10 mm/min was used for the tests.
